# The Nepean Belief Scale (NBS) as a tool to investigate the intensity of beliefs in anorexia nervosa: psychometric properties of the Italian version

**DOI:** 10.1007/s40519-023-01620-w

**Published:** 2023-10-31

**Authors:** Arianna Sciarrillo, Francesco Bevione, Marta Lepora, Federica Toppino, Maria Carla Lacidogna, Nadia Delsedime, Matteo Panero, Matteo Martini, Giovanni Abbate Daga, Antonio Preti

**Affiliations:** Eating Disorders Unit, Department of Neuroscience “Rita Levi Montalcini”, University of Turin, Via Cherasco 15, 10126 Turin, Italy

**Keywords:** Insight, Awareness of illness, Anorexia nervosa/diagnosis, Beliefs, Conviction, Psychometrics/methods, Body mass index

## Abstract

**Background:**

People with anorexia nervosa (AN) show a peculiar impairment of insight regarding their condition, often manifesting a denial of extreme emaciation and sometimes hiding or underreporting socially undesirable abnormal eating patterns. Sometimes the intensity of the beliefs held by patients with AN reach a delusional intensity.

**Objectives:**

In this study, the Italian version of the Nepean Belief Scale was applied to a sample of patients diagnosed with AN to investigate the intensity of their beliefs and convictions and its clinical correlates.

**Methods:**

The Nepean Belief Scale (NBS) was translated and adapted to Italian and applied to a sample of patients diagnosed with AN based on the Structured Clinical Interview for DSM-5 (SCID-5).

**Results:**

The Italian version of the 5-item NBS showed excellent reliability. Convergent validity was proved by negative association with levels of insight measured with the Schedule for the Assessment of Insight in Eating Disorders. Beliefs of delusional intensity were reported by 10% of participants. Those with a greater intensity of beliefs, either overvalued or delusional ideas, were more likely to report poorer general cognitive performances on the Montreal Cognitive Assessment. No association was observed between NBS score and age, body mass index, symptoms of eating disorders, body dissatisfaction, or levels of depression. Fear of weight gain and control seeking were the most often reported themes at the NBS.

**Conclusions:**

The Italian version of the NBS is a reasonably reliable, valid, and usable tool for the multidimensional assessment of insight in AN.

*Level of evidence* Level III, Evidence obtained from well-designed cohort or case–control analytic studies.

**Supplementary Information:**

The online version contains supplementary material available at 10.1007/s40519-023-01620-w.

## Introduction

The concept of belief is not easy to define. At first, a belief can be conceived as a mental attitude or conviction that something is true, real, or exists, without necessarily having proof to support it [1]. Beliefs can derive from personal experiences, or be formed through cultural and social influences [2]. Beliefs are thought to shape a person’s perception of the world, their behavior, and their decision-making processes [3]. Beliefs can play an important role in the development and maintenance of mental disorders [4–7]. In some cases, certain beliefs or cognitive biases can contribute to the onset of mental disorders or make them worse (e.g., [8]). These beliefs can cause significant distress and interfere with the ability to function in daily life [9]. Moreover, people with mental disorders may hold negative beliefs about themselves and their condition due to societal stigma and shame [10, 11]. These beliefs can contribute to feelings of isolation, shame, and reluctance to seek treatment [12, 13].

People diagnosed with anorexia nervosa (AN) may hold irrational beliefs about their body size or weight, sometimes heavily contradictory to the evidence [14]. Often they may deny or minimize the severity of their symptoms, resist treatment, or be unaware of the consequences of their behaviors [15]. In a subgroup of patients diagnosed with AN, the intensity of beliefs about shape and weight may overextend from mild overvalued ideas to overtly delusional intensity [16]. It is unclear how much the deficit of insight, which is described in patients with AN, contributes to the intensity of these irrational beliefs or if it is the ego-syntonic nature of the disorder, leading the patients to adhere to their convictions even when in contrast to other people's concerns [17, 18]. Fear of stigma and the firm willingness to maintain control over their eating behavior may support, too, the intensity of irrational beliefs in AN [19].

Insight is a concept commonly used in psychiatry and refers to a patient’s understanding and acceptance of their mental illness or disorder [20, 21]. It includes recognizing that one has a mental illness, understanding the symptoms and their impact on one’s life, and accepting the need for treatment. Insight and intensity of conviction are related but distinct concepts. In the context of mental illness, insight may involve recognizing that one’s thoughts or behaviors are a symptom of a mental health condition, while the intensity of conviction may refer to how strongly a person believes in the content of those thoughts or behaviors. Overall, both insight and intensity of conviction are important factors to consider in the diagnosis and treatment of a mental disorder, but they should be assessed separately and in the appropriate context. In AN, insight is an important factor and can moderate the severity of certain cognitive and behavioral components of the disorder [22]. Poor insight is associated with later access to treatment and prolonged symptomatology [23]. It is unclear how the deficit of insight in AN is related to the intensity of their irrational beliefs about weight and shape.

With the spreading of interest in illness awareness in psychiatry, several tools have been developed to measure insight in people with mental disorders. Most of these tools have been developed to measure the deficit of insight in psychosis and might be not suited to measure insight in AN [20]. Recently a semi-structured interview has been devised to specifically rate insight in patients diagnosed with an eating disorder, the Schedule for the Assessment of Insight in Eating Disorders-SAI-ED [22]. Evidence of its reliability and validity has been reported in an Italian sample of patients diagnosed with AN (Sciarrillo, submitted manuscript). In the past, the intensity of beliefs in AN has been measured with the Brown Assessment of Beliefs Scale (BABS), which was developed to measure anomalous conviction in people with psychosis [24, 25]. More recently, the Nepean Beliefs Scale (NBS), devised to measure the intensity of beliefs in patients diagnosed with obsessive–compulsive disorder (OCD) [26, 27], has been used to rate the intensity of beliefs in patients with AN [16].

### Aims

This study illustrates the first Italian application of the NBS to patients diagnosed with AN. The aims of this study were: (a) to assess the reliability of the Italian version of the NBS in a sample of patients diagnosed with AN; (b) to ascertain the links between the intensity of beliefs in patients with AN and their degree of insight as measured by the SAI-ED; (c) to evaluate the relationship of the intensity of beliefs with symptoms of eating disorder and of anxiety and depression.

## Methods

This study was approved by the Ethical Committee of the local academic hospital (Prot. n. 0014269; code: 446/2021; signed on February 8th, 2022), and all participants signed written informed consent.

### Participants

All consecutive admissions among those seeking voluntary admission for AN to a major academic Eating Disorders Center between March 2022 and June 2023 were invited to participate in the study.

Participants were included when they were: (a) aged between 18 and 50 years old; (b) had a confirmed diagnosis of AN according to the Structured Clinical Interview for Diagnostic and Statistical Manual of Mental Disorders, 5th edition (SCID-DSM-5); (c) had the capacity of reading the Italian language; (d) had no current or past psychotic or bipolar disorder or current substance use disorder.

### Measures

The study applied the semi-structured NBS interview to the participants (see supplementary material Table S1). Standard translation and back-translation procedures were used to translate the Italian version of the NBS from the English version [28]. Additional tools used in this study were the SAI-ED interview, the Montreal Cognitive Assessment (MoCA), the Eating Disorder Examination Questionnaire (EDE-Q), the Body Shape Questionnaire (BSQ), the State-Trait Anxiety Inventory (STAI); and the Beck Depression Inventory second edition (BDI-II) [29].

The NBS is a five-item semi-structured interview that after the identification of a main belief of the patient, rates five belief characteristics: conviction, fixity, fluctuation, resistance, and awareness that the belief is unreasonable. Each item is rated on a 5-point Likert scale (from 0 to 4). A total score of the intensity of the belief is calculated by summing up all item scores, ranging from 0 to 20 (The Italian version is reproduced in Appendix 1). Internal consistency and inter-rater reliability of the NBS were found good (> 0.70) in past studies on clinical samples [16, 27, 30]. Based on the replies on the BABS in a past study [16], the following thresholds were applied to the scores on the NBS: 0–10, “good/fair insight”; 11–15, “over-valued idea”; 16 or higher, “delusional belief”.

The SAI-ED is a semi-structured interview that measures three major components of insight, such as the ability to recognize that one has a mental illness, the ability to relabel unusual mental events as pathological, and compliance with treatment [22]. The SAI-ED includes two questions on awareness of the illness and the need for treatment, rated on a 0–2 scale; four questions on the recognition of symptoms of eating disorder and their relabeling as attributable to the illness, plus a question on the hypothetical contradiction between own’s view of the condition and concerns of other people, all rated on a 0–4 scale. There is also a final question on treatment adherence, aimed at collecting therapist impressions, rated on a 0–5 scale. The SAI-ED produces two global scores: a subtotal on awareness of illness and symptoms, the sum of the first seven items (ranging from 0 to 24); and a total score, measuring the global level of insight, the sum of all items (ranging from 0 to 29). The reliability of the SAI-ED was reported to be high [22]. In the initial validation study, the lack of insight was likely for a global score on the SAI-ED ≤ 18 [22]. In the Italian validation study, the SAI-ED had moderate reliability (intraclass correlation coefficient [ICC] = 0.71; 95%CI 0.49–0. 86) (Sciarrillo, submitted manuscript).

The MoCA is a widely used cognitive screening tool that is designed to assess various cognitive domains, including attention and concentration, executive functions, memory, language, visuoconstructional skills, conceptual thinking, calculations, and orientation. The MoCA consists of a brief questionnaire and a series of tasks that assess different cognitive ability [31]. It takes approximately 10–15 min to complete. The MoCA is commonly used to screen for cognitive impairment and it is also used in research studies to assess cognitive function and to track changes in cognitive ability over time. The MoCA has acceptable internal consistency (Cronbach’s α > 0.70) [31, 32]. Acceptable inter-rater and intra-rater reliability were reported for the Italian validation, too, when used to detect cognitive impairment [33]. However, the MoCA was developed to be sensitive to mild cognitive impairment in geriatric populations: in healthy people, inter-rater reliability, when measured with the kappa coefficient, varied from 0.46 to 0.94 [34]. This is likely to depend on the ceiling effect, which occurs when a large percentage of participants achieve the highest score on a test. The ceiling effect limits the ability of a test of discriminating subjects at the top level of the performance, thus decreasing the test's reliability, a consequence which is more often observed for cognitive and neuropsychological measures [35].

The EDE-Q is a self-report questionnaire consisting of 28 items that assess four main symptom areas of eating disorders, namely dietary restraint, eating concerns, weight concerns, and shape concerns [36]. Participants are required to rate the intensity of their behavior or thoughts during the last 28 days on a scale ranging from 0 to 6, with a global score also being produced. Clinical samples indicate good reliability of the tool, typically > 0.80, which was confirmed in the Italian validation, too [37].

The BSQ is a 34-item self-report questionnaire specifically aimed at assessing body dissatisfaction prompted by the feelings of being fat [38]. Participants rate their responses on a six‐point scale, with a time interval focused on the past 4 weeks, where higher scores indicate greater dissatisfaction with body shape. The validity and reliability of the BSQ-34 have been proved [39], with reliability judged excellent (around 0.90), as confirmed in the Italian validation [40].

The STAI is a self-report questionnaire comprising 40 items that measure anxiety levels [41]. For the purposes of this study, the version Y of the STAI has been used [42]. Participants rate their levels of anxiety on a scale from 1 (never) to 4 (always) for each item, based on their current state anxiety (20 items) or habitual trait anxiety (20 items). The reliability of the STAI varies from 0.65 to 0.90, depending on the sample [43]. The Italian validation confirmed the psychometric properties of the STAI.

The BDI-II is a self-report questionnaire consisting of 21 items that assess the presence and severity of depressive symptoms [29]. Participants rate their behavior or thoughts on a scale of 0 to 3, with higher scores indicating greater severity of depression symptoms. Scores above 16 are indicative of severe depressive symptomatology. The tool has a high internal consistency, with a reliability of around 0.90 across samples [44]. The reliability and validity of the BDI-II have been confirmed in the Italian version [45].

### Procedure

Participants were individually interviewed with the NBS, SAI-ED, and the MOCA in a quiet room, with the interviews typically lasting an average of 15 min for each task. Furthermore, participants were also requested to complete additional questionnaires through the REDCap platform (Research Electronic Data Capture, Vanderbilt University), using a tablet or smartphone. The time taken to complete these questionnaires was not measured. Prior to the assessment, participants were required to provide informed written consent, and anonymity was ensured.

### Data analysis

Analysis was done with the Statistical Package for the Social Sciences (SPSS) version 28 (IBM Corp. Released 2021) and dedicated packages running in R [46]. The significance threshold was set at *p* < 0.05. All analyses were two-tailed.

Continuous variables were reported as means with standard deviations and range. Skewness and kurtosis were also reported, with values ≥ 2 indicating deviation from the normal distribution according to a conservative threshold for sample size ≤ 50 [47].

Categorical variables were reported as counts and percentages. Analysis of continuous variables was performed with Student’s, or Welch’s t-test in case of violation of the homogeneity of the variance, or with analysis of variance (ANOVA). Categorical variables were analyzed with Chi-square, with Yates correction when necessary. Pearson correlation coefficient was used to measure the association between continuous variables. Effect size in between-group comparisons and correlations was assessed with reference to the thresholds suggested by Cohen [48].

The reliability of the self-report questionnaires was measured with Cronbach’s alpha to facilitate comparisons with previous studies. According to a shared rule, Cronbach’s alpha is assumed to be fair when it is equal to or greater than 0.70 in a questionnaire measuring one latent dimension, good when is greater than 0.80, excellent when exceeds 0.90 [49].

Reliability of the SAI-ED, NBS, and MOCA was assessed as inter-raters agreement with ICC with 95% confidence interval (CI). The calculation was with a two-way random-effects model. According to a shared rule of thumb, ICC values between 0.40 and 0.59 indicate fair reliability; 0.60 to 0.74 indicate moderate reliability; values 0.75 and above indicate excellent reliability [50].

This is a pilot validation study, and sample size requirements were kept to a minimum. For reliability purposes, a sample size of 25/30 participants can be enough to maintain a balance between the power and the precision of reliability estimates [51]. Validity was estimated by correlational analysis. For a two-tailed alpha at 0.05, power at 80%, and for a minimum clinically meaningful effect size of 0.45 (= 20% of shared variance), the required sample size was 33. The calculation was done with G*Power 3.1 [52]. The final sample size in the study was above these minimum requirements.

## Results

Overall, 60 patients were invited to take part in the study. Among them, 11 refused, 4 were too ill to be involved in the study, 1 was discarded because had difficulties in reading the Italian language, and 6 were outside the age cutoff (*n*. = 2 were < 18 years old, and *n*. = 4 were > 50 years old).

The final sample included 38 patients diagnosed with AN, of whom, 28 with AN restricting type (AN-R), and 10 with AN binge-eating/purging type (AN-BP).

Participants included one male (3%) and 37 females (97%), of whom six did not adhere to a binary classification of the genre. Age in the sample ranged from 18 to 49 years, with mean = 24 ± 9. BMI ranged from 11 to 17, with mean = 14 ± 2. Duration of illness ranged from 0.5 year to 35 years, with mean = 7 ± 9.

### Reliability

Inter-rater reliability was fair for MoCA (ICC = 0.53; 95%CI 0.32 to 0.73), and moderate to excellent for SAI-ED (0.74; 0.56–0.86), and NBS (0.80; 0.67–0.89). Cronbach’s alpha for questionnaire varied from good to excellent for EDE-Q (Cronbach’s alpha = 0.93), BSQ (0.97), STAI (0.95), and BDI-II (0.95).

### Scores on the measures used in the study

Table 1 lists the scores for each measure used in the study. No deviation from the normal distribution was observed based on skewness and kurtosis. As for the MoCA, a ceiling effect was found: 9 participants (24%) scored 30, the maximum value on the test, and an additional 8 (21%) scored 29. Overall, 45% of the sample scored at maximum or near maximum value on the MOCA. The high ceiling effect justifies the observation of a relatively low reliability of the test in the sample.

**Table 1:** Mean scores of the measures used in the study in the sample (*n*. = 38)

	Mean (SD)	Range	Skew	Kurtosis
EDE-Q				
Restraint	3.7 (1.8)	0–6	− 0.5	− 0.9
Eating concern	3.1 (1.5)	0.2–5.4	− 0.4	− 0.9
Shape concern	4.3 (1.6)	0.9–6	− 0.6	− 1.00
Weight concern	3.5 (1.9)	0.2–6.0	− 0.3	− 1.3
BSQ	122 (43)	46–187	− 0.2	− 1.2
STAI				
State anxiety	57 (13)	31–77	− 0.6	− 0.2
Trait anxiety	60 (11)	34–74	− 0.8	− 0.4
BDI-II	25.3 (15.9)	3–56	0.5	− 0.9
MoCA	27.2 (2.8)	19–30	− 1.1	0.6
SAI-ED	21.5 (4.5)	12–29	− 0.1	− 0.9
NBS	9.9 (4.9)	2–18	− 0.2	− 1.2

### Intensity of belief at the NBS

The mean beliefs score in the sample for the NBS was 9.9 ± 4.9; range: 2 to 18. Item-total correlation was good (> 0.70), except for item 4 (Resistance). Maximum intensity of the beliefs (score = 4) was rated by less than one-fifth of the sample for each item (Table 2).

**Table 2:** Mean scores of the Nepean Beliefs Scale scores

	Mean	SD	Median	Range	Rated 4	Correlation with total score
Conviction	2.6	1.1	3	0–4	8 (21%)	0.88
Fixity	2.1	1.3	2	0–4	6 (16%)	0.69
Fluctuation	2.2	1.2	2	0–4	6 (16%)	0.79
Resistance	1.7	1.2	1	0–4	2 (5%)	0.51
Reasonableness of belief	1.5	1.5	1	0–4	2 (5%)	0.83

According to the predefined thresholds, 19 (50%) participants showed good/fair insight, 15 (39.5%) overvalued ideas and 4 (10.5%) had a delusional intensity of beliefs. Participants with poor insight at the SAI-ED were 11 (29%).

There was no statistically significant association between these three degrees of belief and categorically rated poor insight: *χ*^2^ = 3.35; *df* = 2; *p* = 0.19. However, several cells have less than 5 counts, thus the result must be taken with caution.

### Association of clinical variables with the intensity of beliefs on the NBS

The intensity of belief, as rated continuously on the NBS, was unrelated to age, BMI, symptoms of eating disorders as rated on the EDE-Q, body dissatisfaction rated on the BSQ, levels of depression on the BDI-II (Table 3).

**Table 3:** Correlations between socio-demographics and clinical features and the Nepean Beliefs Scale total scores in patients diagnosed with anorexia nervosa (n. = 38)

	Nepean Beliefs Scale
Age (years)	*r* = − 0.09; *p* = 0.59
Body mass index (kg/m^2^)	*r* = − 0.09; *p* = 0.59
EDE-Q	
Restraint	*r* = 0.05; *p* = 0.76
Eating concern	*r* = 0.18; *p* = 0.28
Shape concern	*r* = 0.10; *p* = 0.55
Weight concern	*r* = 0.19; *p* = 0.25
BSQ	*r* = 0.09; *p* = 0.59
STAI	
*State anxiety*	*r* = 0.42; *p* < 0.01
*Trait anxiety*	*r* = 0.27; *p* = 0.10
BDI-II	*r* = 0.18; *p* = 0.28
MoCA	*r* = − 0.57; *p* < 0.01
SAI-ED	
Awareness of illness and symptoms	*r* = − 0.46; *p* < 0.01
Adherence to treatment	*r* = − 0.39; *p* = 0.02
Level of insight	*r* = − 0.54; *p* < 0.01

A positive association was observed between state but not trait anxiety and total scores on the NBS. The intensity of beliefs, rated on the NBS, was negatively related to general cognition, as measured with MoCA, and treatment adherence and insight, as measured by the SAI-ED. Put in another way, those with a greater intensity of beliefs, either overvalued or delusional ideas, were more likely to report poorer performances on the MoCA, adhere less to treatment, and be rated with a lower insight.

### Emerging themes during the NBS interview

The self-reported beliefs of the participants that emerged during the interview with the NBS were analyzed to identify any recurring thematic connections. Various themes emerged from the sample, listed in Table 4, including concerns about weight gain (39%), efforts to exert control (21%), perceptions of self-worth (5%), critical self-evaluation (11%), and other related (13%) or multiple/unclassified categories (11%).

**Table 4:** Emerging themes observed in participants' beliefs

Theme	Example	*n*. (%)
Fear of weight gain	*“Adding the legumes to my diet will surely make my weight increase by 1 kg.”*	15 (39%)
Control seeking	*“If I keep under control my diet, then I will consequently take control of all aspects of my life.”*	8 (21%)
Self-worth	*“I’m convinced I’m not thin enough despite my weight.”*	2 (5%)
Negative evaluation	*“My legs are fat and ugly despite what everybody tells me.”*	4 (11%)
Other	*“As more I move, as more my emotions come out and I feel them.”*	5 (13%)
Multiple/unclassified	*“Some foods are damaging to my body, even if nobody told me they are [ex. bread, pasta, floury, fruit].”*	4 (11%)

## Discussion

The results of this pilot study indicate that the Italian version of the NBS shows moderate-to-excellent intra-rater reliability and possesses good concurrent validity, as revealed by the inverse correlation between the intensity of the belief and levels of insight as measured on the SAI-ED. Moreover, NBS scores were negatively related to treatment adherence, confirming that beliefs of particular intensity, assimilable to overvalued or delusional ideas, impair treatment adherence and might negatively affect therapy outcomes.

As already noted, a key advantage of the NBS is the offering of an operational definition of the belief and its characteristics, and this facilitates the accurate recording of beliefs in patient populations [16, 30]. In this study, the mean score for conviction was relatively high—2.6 (see Table 2), and one-fifth circa of patients rated their belief as probably true. Fixity of the belief was on average high, too (mean score = 2.1; median = 2), and one-fifth of patients endorsed their belief as true even against contrary evidence. This was confirmed by relatively high scores on fluctuation, with 16% of the patients holding their belief as true over time. A lower fraction of patients expressed resistance to the belief (5%) or communicate the awareness that their belief was unreasonable (5%). Overall, results were closer to those found in patients with OCD [27] than in patients with psychosis [30].

The percentage of patients with beliefs of delusional intensity (10%) was similar to the one found in past studies with different tools, e.g., 16% in Hartmann et al. [53], 10% in Mountjoy et al. [54], but lower to the percentage observed in a past study with the NBS (23.7%) [16] or when measured with BABS (e.g., 20% in [25]; 22.7% in [22]). Discrepancies might depend on some unmeasured characteristics of the samples, such as psychiatric or somatic comorbidity, which was rarely or never reported in past studies. Indeed, in research, psychiatric comorbidity should be better investigated as clinicians often tend to underdiagnose the present clinical pictures that are different from ED [55].

In this study, MoCA scores were negatively related to scores on the NBS, thus indicating that as poorer the cognitive performances on the MoCA, as greater the chances of finding delusional intensities on the NBS. In patients with psychosis, the NBS showed good convergent validity with the BABS [30]. Patients with schizophrenia-spectrum psychosis are known to display cognitive impairment on the MoCA [56]. Comorbidity of AN with psychosis has long been debated [57–59], but some features such as rigidity of thought [60, 61], abnormal bodily perception [62], and hallucinations-like experiences such as the “anorexic voice” [63], might be assimilated to psychotic-like traits. Moreover, the phenotypic relationships between eating disorders and psychosis seem supported by shared genetic vulnerability [64], as confirmed by their familial co-aggregation [65]. The fraction of patients with AN also displaying psychotic traits might influence the finding of delusional intensities on the NBS. However, the negative association of NBS scores with a measure of general cognition might depend on the negative impact of malnutrition on cognition, albeit we did not find an association of BMI with NBS scores.

As in past studies [16, 53], we did not find a relationship between body dissatisfaction and intensity of beliefs in our sample. Instead, we found a link between trait anxiety and the intensity of beliefs on the NBS. This might suggest that those with overvalued or delusional ideas experience greater anxiety because of their beliefs, thus making them less ready to have their highly regarded beliefs challenged during treatment. On a therapeutic ground, this is important since the primary treatment of delusional ideas is with antipsychotics, and the efficacy of antipsychotics such as olanzapine or aripiprazole in the reduction of delusional beliefs in AN has yet to be established [66, 67].

### Strengths and limitations of the study

The use of two interviews to investigate beliefs and insight in the sample is the most significant strength of the study since self-report tools are more exposed than interviews to misunderstanding, hiding, and social desirability replies. It must be considered that while the SAI-ED has been developed specifically for patients with eating disorders, the NBS has been devised for use in patients with obsessive–compulsive disorder. Nevertheless, its application in this and a past study [16] indicates that the tool can be applied to patients with AN. It is worth noting that the Italian validation of the SAI-ED has not yet been published and that the Italian NBS has not been validated so far in people with obsessive–compulsive disorder (OCD), who were the original clinical target of the tool. Thus, evidence on the reliability and validity of the Italian NBS in patients with AN should not be extrapolated to Italian patients with OCD before appropriate testing.

Some limitations have to be taken into account, too. The sample size was not large enough to allow more complex statistical analyses, e.g., a comparison between AN-R and AN-BP participants. The small sample size might also have prevented the detection of a statistically significant association between the three degrees of belief drawn from the NBS and categorically rated poor insight on the SAI-ED. We could not address the role of psychiatric or somatic comorbidity in the sample since information was limited. Participants included help-seeking individuals requiring inpatient treatment, which implies some insight among them. Moreover, the most severe patients were not recruited for ethical reasons. The sample cannot be considered completely representative of the population from which it was drawn. All but one patient were women. Therefore it cannot be assured that the findings will hold in male patients with AN.

### What is already known on this subject?

Insight and intensity of beliefs about weight and body concerns influence adherence to treatment and outcome in patients with AN [15, 19, 23]. The NBS has been devised to measure intensity of beliefs in patients with obsessive–compulsive disorder as a proxy of insight [27]. NBS was proved to be applied to patients with psychosis, too, a population with difficulties in achieving awareness of illness and insight about it [30]. Overall, the NBS is an agile tool that can be applied in the short term with adequate reliability, a clear advantage in patients that may be challenging to be evaluated.

### What does this study add?

The Italian version of the NBS showed good psychometric properties in terms of reliability and concurrent and divergent validity. The findings of this study confirm and extend the results of a past application of the NBS to a sample of patients with AN [16]. In this population, the NBS allows the identification of a subgroup of patients with beliefs of delusional intensity.

## Conclusions

While it is still unknown the prognosis of those patients with AN that nurture delusional beliefs, their identification is helpful for therapeutic purposes. Future studies should test whether there are differences across subtypes of AN in terms of the intensity of beliefs, and evaluate which relevant factors influence the occurrence of beliefs with delusional intensity. The sensitivity of delusional beliefs in patients with AN to treatment with antipsychotics should be explored with randomized-controlled trials.

### Supplementary Information

Below is the link to the electronic supplementary material.

Supplementary file 1 (PDF 167 KB)

## Data Availability

The corresponding author had full access to all the data in the study and takes responsibility for the integrity of the data and the accuracy of data analysis. Data sharing is not applicable since consent to publish was for aggregated data only.

## Code availability

Standard codes were used in the analysis. Sample codes will be shared upon demand.
