# Supplementary material for: The Nepean Belief Scale (NBS) as a tool to investigate the intensity of beliefs in anorexia nervosa: psychometric properties of the Italian version
Source: Eat Weight Disord. 2023 Oct 31;28(1):92. doi: 10.1007/s40519-023-01620-w (PMC10618389; doi:10.1007/s40519-023-01620-w)
Supplement: Supplementary file 1 — (PDF 167 KB) [file 40519_2023_1620_MOESM1_ESM.pdf]

NEPEAN BELIEF SCALE

ID: ………………………………………………………………………
Data dell'intervista/colloquio: ……………………….

Descrizione del convincimento
Il convincimento è considerato una sensazione o una credenza che qualcosa esista o sia vero, senza avere la piena conoscenza intellettuale che ne garantisca la verità/realtà.

Nell'ultima settimana, qual è stato il maggiore convincimento associato al sintomo più significativo, doloroso o disabilitante tra i suoi sintomi?

Annotare
_ La scelta di termini e delle espressioni dovrebbe essere verificata con il soggetto/candidato e incorporata nelle domande utilizzate in questa scala.
_ La scelta di termini e delle espressioni dovrebbe anche mirare a essere il più specifica possibile, ad es. invece di "pulisco eccessivamente", "pulisco eccessivamente perché tutto nel mondo è contaminato e potrebbe farmi molto male" (se così si sono espressi i pazienti).
_ È importante che il convincimento individuato possegga una vividezza e una vivacità tali da renderlo chiaramente distinguibile da un'idea o da un assunto.
_ Il convincimento deve inoltre apparire strutturato e persistente, a differenza della natura fluttuante di un pensiero.
____
____
____
____
____

1. Convinzione
La convinzione rappresenta quanto una persona ritenga che il suo convincimento sia vero.

Quanto ritiene che il suo convincimento sia vero?

Il soggetto ritiene che il suo convincimento sia:
0 – Completamente non vero. Assegnare il punteggio di 0 all'*item* 2 e passare direttamente all'*item* 3.
1 – Probabilmente non vero
2 – Possibile, ma resta incerto/insicuro
3 – Probabilmente vero
4 – Assolutamente vero

2. Fissazione
La fissazione è la tendenza a non modificare il convincimento anche quando alla persona sono portate prove incontrovertibili contrarie al suo convincimento.

Se le fosse presentata una prova incontrovertibile che le suggerisce che il suo convincimento è sbagliato, cosa accadrebbe?

Il soggetto penserebbe che il suo convincimento sia:
0 – Completamente non vero
1 – Probabilmente non vero
2 – Possibile, ma resta incerto/insicuro
3 – Probabilmente vero
4 – Assolutamente vero

---

3. Fluttuazione
Con fluttuazione si intendono spontanei cambiamenti nel livello di convinzione, anche quando alla persona non vengono presentate prove contrarie al suo convincimento.

Alcune persone sostengono che i propri convincimenti possano cambiare senza una ragione particolare. Ci sono state occasioni in cui il suo convincimento si è modificato senza particolari ragioni?
Se SÌ, chiedere: *provi a ripensare all'ultima volta in cui un suo convincimento è cambiato senza una ragione particolare. Quanto riteneva che il suo convincimento fosse vero in quel momento?*
Se NO, quantificare quanto il paziente creda che il suo convincimento sia vero (come nell'*item* 1).

Il soggetto riteneva che il suo convincimento fosse:
0 – Completamente non vero
1 – Probabilmente non vero
2 – Possibile, ma resta incerto/insicuro
3 – Probabilmente vero
4 – Assolutamente vero

---

4. Resistenza
La resistenza è lo sforzo che la persona applica per allontanare il suo convincimento; ciò comporta che la persona si senta a disagio o stressata a causa del suo convincimento o che lo ritenga irrazionale (ad esempio, percepire che il convincimento sia estraneo o "ego-distonico").

Quanto cerca di allontanare il convincimento o smettere di pensare ad esso?

Il soggetto resiste:
0 – Sempre
1 – Frequentemente
2 – A volte
3 – Raramente
4 – Mai

---

5. Consapevolezza che il convincimento sia irragionevole
Il grado di consapevolezza nutrito dalla persona che il proprio convincimento sia irragionevole, inesatto e/o senza senso.

Ritiene che il suo convincimento sia ragionevole o irragionevole?

Il soggetto pensa che il suo convincimento sia:
0 – Completamente irragionevole
1 – Probabilmente irragionevole
2 – Non sa/Non è sicuro
3 – Probabilmente ragionevole
4 – Completamente ragionevole

Punteggio totale (sommare i punteggi degli items 1-5):

Tanto più alto è il punteggio totale dato dalla somma delle singole valutazioni, tanto maggiore è l'intensità del convincimento

Traduzione: Arianna Sciarrillo, Stefano Bonesi, Antonio Preti – 2021

Versione originale: Brakoulias V, Starcevic V, Milicevic D, Hannan A, Viswasam K, Brown C. The Nepean Belief Scale: preliminary reliability and validity in obsessive-compulsive disorder. Int J Psychiatry Clin Pract. 2018 Jun;22(2):84-88. doi: 10.1080/13651501.2017.1374413. Epub 2017 Sep 8. PMID: 28885070.
